# A Preliminary Analysis of the Influence of Elderberry Juice Consumption on Thyroid Metabolism in Mice and Humans Fed High-Fat Diets

**DOI:** 10.3390/nu17040612

**Published:** 2025-02-08

**Authors:** Catherine L. Jarrett, Christy Teets, Franck G. Carbonero, Andrea J. Etter, Patrick M. Solverson

**Affiliations:** 1 Department of Nutrition and Exercise Physiology, Elson S. Floyd College of Medicine, Washington State University, Spokane, WA 99202, USA; catherine.jarrett@wsu.edu (C.L.J.); christy.teets@wsu.edu (C.T.); franck.carbonero@wsu.edu (F.G.C.); 2 Department of Nutrition and Food Science, University of Vermont, Burlington, VT 05405, USA; andrea.etter@uvm.edu

**Keywords:** berry anthocyanins, elderberry, functional foods, thyroid metabolism

## Abstract

Background/Objectives: Elderberry juice (EBJ) consumption prevents weight gain in mice fed a high-fat diet and increases fat oxidation in response to a meal challenge in overweight humans. Thyroid hormones influence metabolism and substrate oxidation, and the impact of EBJ consumption on thyroid homeostasis remains unexplored. Thus, the primary objective of this analysis was to investigate whether elderberry consumption in mice and humans affects serum thyroid biomarkers. Methods: Serum samples from a previous trial incorporating an EBJ intervention were analyzed for thyroxine (T4), thyroid stimulating hormone (TSH), and thyroglobulin (Tg). The samples are from a meal-tolerance test in 18 humans who followed a 7-day diet-controlled crossover design. Samples from 33 male mice were collected after 13 weeks following a high-fat diet with or without EBJ powder, and with or without free wheel running. Results: Short-term EBJ consumption in humans resulted in significant increases in T4 (PL: 42.3 ± 3.2 vs. EBJ: 49.3 ± 4.1 ng/mL, *p* < 0.05), and TSH (PL: 0.094 ± 0.012 vs. EBJ: 0.104 ± 0.011 ng/mL, *p* < 0.05), with no change in Tg (*p* > 0.05). Whereas supplementation with EBJ powder in rodents resulted in a non-significant reduction in T4 (*p* = 0.07). Conclusions: These findings suggest that elderberry juice consumption may influence thyroid metabolism, contributing to the observed metabolic benefits, such as improved fat oxidation, body composition, and protection against high-fat diet-induced weight gain. The increased T4 and TSH in humans align with enhanced metabolic rate, while the reduction in T4 in rodents indicates potential long-term adaptations requiring further exploration.

## 1. Introduction

Elderberries are an emerging specialty crop gaining in popularity as a health food. This is in part due to the nutritional composition, and bioactive properties. Elderberry juice (EBJ) consumption has been shown to support gut health, improve glucose metabolism, lower inflammation, and influence fat metabolism [1,2,3,4,5]. Recent studies from our group demonstrate that both acute and chronic elderberry consumption can prevent weight gain in mice fed a high-fat diet and increase fat oxidation in response to a meal challenge in overweight humans [1,2].

Thyroid hormones play a part in the modulation of lipid metabolism and metabolic rate, which influences body weight regulation and composition. Evidence from a high-fat feeding study in male mice suggests that obesity-prone mice exhibit increased hypothalamus–pituitary–thyroid (HPT) axis activity. Whereas obesity-resistant mice maintain body weight and exhibit elevated deiodinase 1 enzyme activity, which regulates thyroid hormone availability [6].

Vitamins and polyphenols, especially anthocyanins, are key bioactive nutrients in elderberries that may impact thyroid function and play a role in weight gain prevention and improved fat oxidation. The data regarding these important bioactive nutrients on thyroid function are mixed and limited. For example, a cross-sectional analysis of adults in the US has shown that vitamin C intake is associated with lower total thyroxine (T4) but not thyroid-stimulating hormone (TSH) levels [7]. A 6-month randomized trial in older adults found that grape extract supplementation, which is high in polyphenols, resulted in no changes in T4 or TSH levels [8]. Interestingly, when middle-aged women consumed a polyphenol-rich fruit juice for 10 weeks, providing 194 mg of anthocyanins, juice consumption increased urine thyroxine excretion and improved cognitive function [9]. Cognitive improvement and thyroxine modulation by polyphenols may be explained by the ability of select members to interact with transthyretin [10].

Recognizing the significance of elderberries as a dietary supplement crop, the U.S. Department of Agriculture recently funded a program to advance the agricultural development of the dietary supplement market (MO-HAPS0004) [11]. This highlights the importance of gaining a better understanding of how elderberries influence metabolism to prevent weight gain and enhance fat oxidation. An unexpected finding from our rodent study was that mice fed EBJ powder consumed more total calories and maintained a lower body weight and fat mass compared to the control, which is suggestive of basal metabolic rate changes. Despite the growing body of evidence linking elderberry consumption to metabolic health benefits, the potential role of anthocyanin-rich elderberries in modulating thyroid hormone has not yet been explored. Therefore, the primary purpose of this retrospective analysis was to investigate whether elderberry consumption in mice and humans affects serum thyroid biomarkers.

## 2. Materials and Methods

### 2.1. Human Clinical Trial and Rodent Study Design

The details and main outcomes of this project were previously published [1,2]. In brief, for the rodent feeding study, 40 four-week-old male mice (*n* = 10 per group) were randomly assigned to one or four conditions (45% high-fat diet with or without 10% elderberry juice powder, denoted HFD or EBJHFD, and exercise or no-exercise condition) for 13 weeks. The human clinical trial (NCT 06626373) included 18 overweight and obese adults (15 female) who completed a 7-day diet-controlled crossover design trial with either twice-daily elderberry juice consumption (355 g of 100% EBJ) or placebo (355 g of color and taste-matched beverage, 11% sugar) with 4 days of 100% investigator-controlled diet periods separated by a 3-week washout before crossover. Study volunteers were instructed to maintain their habitual physical activities throughout the study. They also completed daily questionnaires and returned empty food and beverage containers to the study center to monitor compliance with the protocol. For complete details of diet design, we refer the reader to the original publication [2]. On the morning of the 8th day for each diet period, a 3 h meal tolerance test (MTT) was administered. The MTT consisted of a high-carbohydrate breakfast containing ~60 g of sugar from waffles and syrup, plus elderberry juice or placebo beverage, and serum collections were performed every 30 min for 3 h. The two studies were approved by the Washington State University Institutional Animal Care and Use Committee (WSU-ASAF 7089) and Institutional Review Board (WSU-IRB 18597), respectively. Informed consent was obtained from all human subjects involved and the clinical trial was conducted according to the guidelines of the Declaration of Helsinki.

### 2.2. Human Serum T4, TSH, Tg Quantification

To evaluate thyroid hormones, fasting blood samples (collected before the first bite on test day) and postprandial blood samples (collected 120 min after the challenge meal) were obtained at each treatment endpoint. Blood was collected, clotted, centrifuged, aliquoted, and stored at −80 °C until analysis. Thyroxine (T4) was measured using the Thermo Fisher Thyroxine (T4) Competitive ELISA Kit (Thermo Fisher, Waltham, MA, USA, EIAT4C), Thyroid Stimulating Hormone (TSH) was measured using the Millipore Human Thyroid Stimulating Hormone ELISA Kit (Millipore Sigma, Burlington, MA, USA, RAB0502), and Thyroglobulin (Tg) was measured using the Millipore Human Thyroglobulin ELISA Kit (Millipore Sigma, Burlington, MA, USA, RAB0458). Standards and samples were assayed in duplicate, and absorbance was measured at 450 nm using a BioTek SYNERGY H1 microplate reader (Agilent, Santa Clara, CA, USA).

### 2.3. Mouse Serum T4 Quantification

At the end of the 13-week study, after a 4 h fast, mice were deeply anesthetized via isoflurane and sacrificed by exsanguination via cardiac puncture. Blood was collected, allowed to clot, and centrifuged to isolate serum, which was stored at −80 °C until further analysis. Thyroxine (T4) concentrations in serum samples were measured using a Mouse Thyroxine ELISA Kit (Novus Biologicals, Centennial, CO, USA, NBP2-60162), following the manufacturer’s instructions. Seven of the forty serum samples were hemolyzed upon collection, and T4 quantification was not possible. Quantification of the remaining two thyroid hormones was not possible due to an exhausted serum biobank.

### 2.4. Statistical Analysis

Our approach to statistical testing for both the human and rodent studies is described in detail elsewhere and all modeling was performed in SAS version 9.4 (Cary, NC, USA) [1,2]. Briefly, in the human clinical trial, linear mixed modeling was used to test for statistically significant differences between EBJ and placebo beverage treatments for serum TSH, T4, and Tg, using PROC MIXED, by using SAS version 9.4 (SAS institute, Cary, NC, USA). In addition to testing for the main effects of beverage treatment (EBJ or placebo), time (fasted or 2 h postprandial), and the treatment by time interaction, covariates including volunteer sex, BMI, and age were also tested. Non-significant interactions and covariates were removed from preliminary models by backward elimination. In the rodent feeding study, the main effects of diet, exercise, and their interaction were tested for serum T4 with two-way ANOVA (diet and exercise) with backward elimination of non-significant interactions. Normality was formally tested using Shapiro–Wilk, and non-normality was corrected with mathematical transformation. When reporting group-wise differences, Tukey’s post hoc HSD correction is applied. Data are reported as group means ± SEM, and statistical significance is considered at *p* < 0.05. The de-identified raw data are provided in Supplementary Tables S1 and S2.

## 3. Results

### 3.1. Human Thyroid Hormone Levels

**Figure 1:**
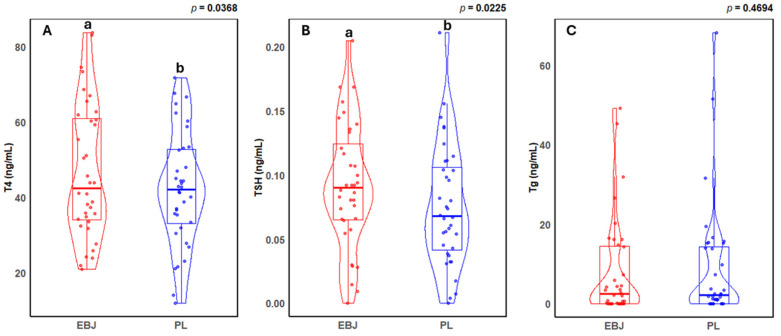
Violin plots with boxplot overlays showing concentrations of thyroid hormones (**A**) T4 (ng/mL), (**B**) TSH (ng/mL), and (**C**) Tg (ng/mL). The results compare the effects of EBJ and PL treatments in humans. EBJ, elderberry juice treatment; PL, placebo treatment; T4, thyroxine; TSH, thyroid stimulating hormone; Tg, thyroglobulin. Pair-wise comparisons without a common letter are significantly different, *p* < 0.05.

In comparing elderberry juice or placebo beverages, a main effect of diet was observed for T4 and TSH, where the elderberry juice beverage caused a ~11% increase in T4 and 21% increase in TSH compared to placebo beverage (*p* = 0.037 and 0.023, respectively). No change in Tg was observed (*p* = 0.47). Data are reported in Table 1 and Figure 1.

### 3.2. Mouse Thyroid Hormone Levels

**Figure 2:**
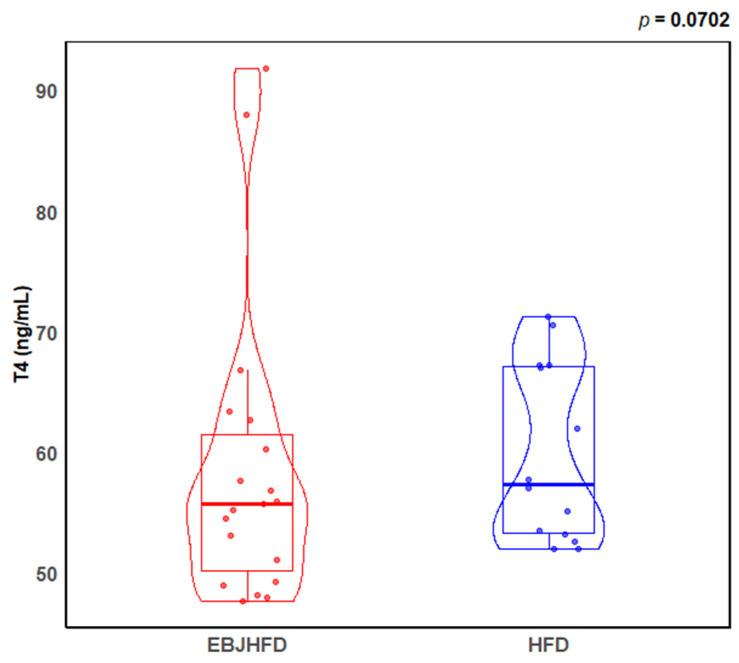
Violin plots with boxplot overlays showing concentrations of thyroid hormones T4 (ng/mL). The results compare the effects of EBJHFD and HFD treatments in mice. EBJHFD, elderberry juice + high-fat diet; HFD, high-fat diet; T4, thyroxine.

After 13 weeks of elderberry consumption there was a non-significant 8% reduction in T4 (*p* = 0.07) compared to placebo. Data are displayed in Table 2 and Figure 2.

## 4. Discussion

The objective of this study was to explore the possible effects of elderberry juice interventions on thyroid metabolism, as our previous findings in both a rodent model of diet-induced obesity and a one-week randomized controlled trial in humans suggest protection against a high-fat diet [1,2]. The 13-week rodent study illustrated the potency of EBJ, where blood glucose metabolism, body weight and composition, and the fecal microbiota were all protected from the metabolic and clinical aberrations of a 45% fat diet when 10% freeze-dried EBJ powder was included. The one-week human trial supported the rodent findings with positive effects of twice-daily EBJ on meal tolerance testing and substrate oxidation compared to a placebo beverage. Unexpectedly, the rodents consuming EBJ powder ate significantly more calories compared to the control group despite maintenance of a lower body weight and fat mass, which suggests modulation of basal metabolic rate. Thus, here we sought to test differences in serum TSH, T4, and Tg between treatment groups in both experiments to determine if the changes noted in metabolic and clinical measures can be partially explained by changes in thyroid metabolism.

All three serum thyroid biomarkers were measured from the human trial, but due to limitations on sample availability only T4 was measured from the rodent obesity experiment. In the human EBJ trial, significant 21- and 11% increases were observed in TSH and T4, respectively, where Tg was unchanged between diet treatments. In the rodent study, serum T4 was only marginally different between diet treatments, where EBJ treatment decreased T4 by 8% compared to the HFD control group.

The differences between elderberry and control treatment groups and discord between a short-term human study and long-term rodent feeding study offer novel discussion on the potential mechanisms of action of berry anthocyanins within the context of a high-fat diet and metabolic rate. Indeed, increased circulating T4 does correspond with increased metabolic rate in humans [12]. In 10 healthy men, 200 micrograms of T4 per day for 3 days increased metabolic rate by 4% and reduced mitochondrial efficiency by 30% in vastus lateralis biopsies. Similarly, anthocyanins are reported to interact with the mitochondrial membrane, promote mitochondrial biogenesis, and increase uncoupling protein 1 expression [13,14,15,16,17].

Several micronutrients in the diet, and the gut microbiome, both influence thyroid function in humans; these relationships have been recently summarized [18]. Moreover, modulation of thyroid function by total flavonoid intake was observed in a retrospective analysis of the National Health and Nutrition Examination Survey (NHANES) 2007-2010 [19]. However, there is scant information on the influence of anthocyanins on thyroid metabolism; any discussion currently available in the scientific literature is limited to its effects on thyroid injuries/cancer. Therefore, increased TSH and T4 in humans consuming EBJ for 1 week adds novelty to the current anthocyanin bioactivity framework, suggesting its action also includes hormonal influence in addition to direct effects on target tissues. Further, the absence of effect on serum Tg concentrations suggests any influence of EBJ on thyroid hormone metabolism is not a stressor to the thyroid gland. The tissues and organs where EBJ anthocyanins and metabolites exert their influence deserve further elucidation.

The discovery of lower T4 in rodents fed an HFD with 10% EBJ powder compared to HFD control is the opposite of the human trial and suggests adaptation that also involves thyroid metabolism. In a study of euthyroid and thyroid-damaged rats fed control or high-fat diets of varying composition for 8 weeks, higher circulating triiodothyronine (T3) was noted in the thyroid-damaged groups and was accompanied by decreased food intake [20]. Conversely, a separate study demonstrated how rats fed HFD for 8 weeks increased metabolic rate and fat oxidation despite no change in circulating T3 or T4 [21]. In the latter study, increased hepatic and renal deiodinase activity may explain changes in metabolism despite unchanged serum thyroid hormones. Interestingly, they also recorded a 30% decrease in nighttime physical activity in the HFD-fed rodents, whereas in our rodent experiment physical activity (measured with metered running wheels) was not different between EBJ and control HFD groups. Therefore, our results suggest differences in rodent T4 after 13 weeks of HFD feeding may have biological significance, as body composition was improved despite no change in physical activity and increased food intake. However, our findings are only suggestive. Taken together, a clearer understanding of the influence of EBJ on human and rodent thyroid metabolism needs to include an investigation into other biomarkers of thyroid status. For example, future studies could consider testing T3, free vs. bound T4 and T3, hepatic and renal deiodinase activities, and leptin, with accompanying measurement of indirect calorimetry to assess prospective changes to metabolic rate [22]. Including these markers in a prospective analysis of short-term and chronic EBJ feeding will help elucidate any potential adaptation of thyroid metabolism and subsequent effects on energy expenditure as suggested by these preliminary findings.

This secondary analysis had many strengths and weaknesses. A key strength of this study is the use of a crossover design in the human trial. Additionally, the human study incorporated dietary control, ensuring the observed effects could be more confidently attributed to EBJ consumption. The rodent study included exercise as a factor, demonstrating that the metabolic benefits of EBJ were independent of physical activity level.

This study has several limitations. It does not provide information on the long-term effects of elderberry juice consumption in humans. The human findings are based on a small sample from a rigorous study, primarily consisting of middle-aged women, which limits the generalizability of the results.

The ability to interpret thyroid function is limited when relying solely on serum levels of T4, TSH, and Tg, as these measurements provide an incomplete picture of the hypothalamus–pituitary–thyroid axis and do not fully capture thyroid hormone signaling or tissue-specific activity. Thyroid hormone levels in plasma are typically stable in healthy individuals; thus, our detection of subtle changes in hormone concentrations may not necessarily translate to changes in metabolic function or activity. However, the present findings are supported by the bioactive properties of EBJ described in our earlier reports. The exact mechanism(s) through which elderberries alter thyroid function and influence metabolism remain unclear, warranting further investigation.

## 5. Conclusions

In conclusion, these findings suggest that elderberry juice consumption may influence thyroid metabolism, potentially contributing to the observed metabolic benefits, such as improved fat oxidation, body composition, and protection against high-fat diet-induced weight gain. The increased T4 and TSH in humans aligns with an enhanced metabolic rate, while the reduction in T4 in rodents indicates potential long-term adaptations requiring further exploration. Elderberries are rich in antioxidants, including vitamin C, vitamin A, and anthocyanins. However, it is difficult to determine which component is driving any changes in thyroid hormone levels or thyroid signaling. These effects could result from a direct interaction or be mediated through the fruit’s anti-inflammatory, metabolic, and prebiotic properties. This work expands the current understanding of elderberries as a functional food, offering evidence of hormonal influence alongside direct metabolic effect, but underscores the need for future investigation.

## Figures and Tables

**Table 1:** Human serum thyroid biomarker concentrations by treatment.

	EBJ	PL	*p*
T4 (ng/mL)	47.0 ± 2.97	42.19 ± 2.51	0.037
TSH (ng/mL)	0.092 ± 0.008	0.076 ± 0.008	0.023
Tg (ng/mL)	11.72 ± 2.76 ^a^	12.23 ± 3.18 ^b^	0.469

Data are expressed as mean ± SEM. *n* = 36 unless otherwise stated; ^a^, *n* = 25; ^b^, *n* = 26; EBJ, elderberry juice treatment; PL, placebo treatment; T4, thyroxine; TSH, thyroid stimulating hormone, Tg, thyroglobulin.

**Table 2:** Summary of mouse thyroid biomarker T4 by treatment.

	EBJHFD	HFD	*p*
T4 (ng/mL)	55.0 ± 1.42 ^a^	59.9 ± 1.96 ^b^	0.070

Data were expressed as mean ± SEM. ^a^, *n* = 14, ^b^, *n* = 17. EBJHFD, elderberry juice + high fat diet; HFD, high fat diet; T4, thyroxine.

## Data Availability

De-identified research data from measures of human and mouse serum thyroid biomarkers are available in Supplementary Tables S1 and S2 described above.

## Supplementary Materials

The following supporting information can be downloaded at: https://www.mdpi.com/article/10.3390/nu17040612/s1, Table S1: human serum thyroid biomarkers; Table S2: mouse serum thyroxine.
